# How to Examine for Movable Kidney

**Published:** 1901-12-21

**Authors:** 


### HOW TO EXAMINE FOR MOVABLE KIDNEY.

At a recent meeting of the Medical Society Mr. Henry Morris detailed very fully the symptoms and the treatment of movable kidney. He first described his method of examining for the discovery of this condition. The patient should be on his back with the shoulders slightly raised by a firm pillow, the spine resting quite flat upon the sofa or mattress, with the lower limbs very slightly flexed. He should lie close to the right edge of the couch for the examination of the right kidney, and close to the left edge for the left kidney. The surgeon then places two fingers of one hand behind and upon, and also just below the last two ribs, and the fingers of the other hand flat upon the front of the abdomen just below the costal margin near the outer border of the rectus muscle. He should be on the same side of the patient as the kidney he is examining, and he should sit or kneel at the bedside so as to have his lowermost forearm as nearly as possible level with the patient's body. The hand on the loin should gently but firmly press forwards the lumbar parietes and should sustain them in that position, and then the fingers of the hand in front should gently and steadily depress the anterior abdominal walls upwards, backwards, and outwards. The kidney, if enlarged or displaced at all, will now be felt between the two hands, and even if not enlarged, the lower pole will, in some persons, be detected by the fingers during natural respiration. If not the patient should take a long and deep inspiration, and suddenly, but without muscular effort, allow the air to escape from his lungs ; in other words, he should make a long-drawn "sigh." The hand behind still maintaining forward pressure on the loin, the hand in front quickly follows the receding abdominal wall as it becomes relaxed during the expiratory movement and the kidney, which otherwise cannot normally be felt, can often then be seized between the fingers of the two hands before it has time to gain its normal position. In testing the mobility of the kidney it is sometimes advantageous to place the patient in the knee elbow or in the upright position, but as a rule it is best to get him to roll over on the opposite side and then to employ bimanual pressure as above described, together with deep inspiration. In this method of examination the kidney is best felt at the end of the inspiration and at the very beginning of expiration, as soon that is as the muscles of the abdominal walls relax.

